# Persistent pulmonary hypertension, neonatal stroke, coagulopathy and multi-organ failure due to severe enterovirus sepsis: two case reports

**DOI:** 10.1186/s12887-026-06812-8

**Published:** 2026-04-07

**Authors:** Arangan Kirubakaran, Kirsty Gray, Thomas Slater, Zoe Cass-Tansey, Amna Alvi, Sundar Sathiyamurthy, Ujwal Kariholu

**Affiliations:** 1 Present Address: Department of Neonatology, Imperial College Healthcare NHS Trust, London, UK; 2 Present Address: Imperial College London, London, UK; 3 Department of Paediatrics, The Hillingdon Hospitals NHS Foundation Trust, London, UK

**Keywords:** Neonatal enterovirus, Persistent pulmonary hypertension, Neonatal stroke, Neonatal coagulopathy

## Abstract

**Background:**

Disseminated neonatal enteroviral infection is a rare and life-threatening condition with significant multi-organ involvement. It often presents with nonspecific symptoms that may be mistakenly attributed to bacterial infections. Prompt recognition and treatment are crucial to prevent rapid disease progression. We report two cases of disseminated neonatal enterovirus, including a novel instance of persistent pulmonary hypertension (PPHN) and neonatal stroke.

**Case presentation:**

A baby boy was delivered vaginally at 35 + 4 weeks, complicated by meconium-stained liquor. He developed respiratory distress after birth, requiring intubation. Subsequently, he developed PPHN, pneumothorax, hypotension, hepatic dysfunction, coagulopathy, and a haemorrhagic stroke, culminating in a decision to re-direct care. He died on day 6 of life. He tested positive for enterovirus from his salivary swab and serum PCR.

A baby boy born at 37+2 weeks was delivered in good condition by elective Caesarean section. He was incidentally found to exhibit respiratory distress on day 5 of life, with the rapid development of coagulopathy, hypotension and seizures shortly afterwards. He tested positive for enterovirus on both salivary swabs and serum PCR samples. He achieved a complete clinical recovery and was subsequently discharged home. He continues to remain well at follow-up.

**Conclusions:**

Hypoxia, coagulopathy and hepatic or metabolic derangement that are inconsistent with the clinical history should raise suspicion of enteroviral infection. Haemorrhagic stroke and PPHN should also be considered as additional manifestations of disseminated enteroviral infection. Prompt recognition can facilitate the administration of intravenous immunoglobulin or antiviral therapy, which may improve survival.

## Introduction

Enteroviruses are RNA viruses belonging to the *Picornaviridae* family, encompassing viruses such as echovirus and coxsackievirus A or B. These viruses are common causes of self-limiting illnesses often mistaken for bacterial infections in children. Severe infections during the neonatal period are rare, with an estimated mortality rate of 30.4% based on 237 cases documented in the literature [1]. Complications of enteroviral infections include myocarditis, hepatitis, coagulopathy, and encephalitis.

Maternal infection is common, with a longitudinal survey showing that enteroviral infections affect approximately 42% of pregnant women [2]. Diagnosis is typically confirmed through polymerase chain reaction (PCR) analysis of stool or respiratory specimens.

We present two cases of enteroviral infections with distinct manifestations and varied outcomes. The first case involved persistent pulmonary hypertension, neonatal stroke, progressive hepatic and renal failure, while the second case featured progressive coagulopathy necessitating multiple blood product transfusions.

## Case A

A male infant weighing 2407g was born at 35 + 4 weeks gestation following a spontaneous onset of labour and vaginal delivery complicated by meconium-stained liquor. He cried immediately after birth but soon developed grunting and persistently low saturations requiring up to 100% oxygen via a T-piece circuit by 15 min of life. Due to hypoxia combined with prematurity and meconium-stained liquor, he was intubated and received 480 mg of surfactant (poractant alfa), which improved his oxygen saturations before transfer to the neonatal unit. His Apgar scores were 8 at 1 min, 7 at 5 min and 7 at 10 min.

Immediately after admission to the neonatal unit, his respiratory support was escalated to high-frequency oscillatory ventilation (HFOV) and inhaled nitric oxide therapy was initiated due to presumed persistent pulmonary hypertension of the newborn (PPHN). He received a 10 ml/kg transfusion of packed red blood cells to optimise treatment in response to hypoxic respiratory failure.

Umbilical venous and arterial lines were inserted to facilitate inotropic support. He experienced a sudden brief cardiac arrest associated with a right-sided pneumothorax. Cardiopulmonary resuscitation was commenced, involving two doses of adrenaline and sodium bicarbonate, immediate needle decompression and chest drain insertion.

**Fig. 1:**
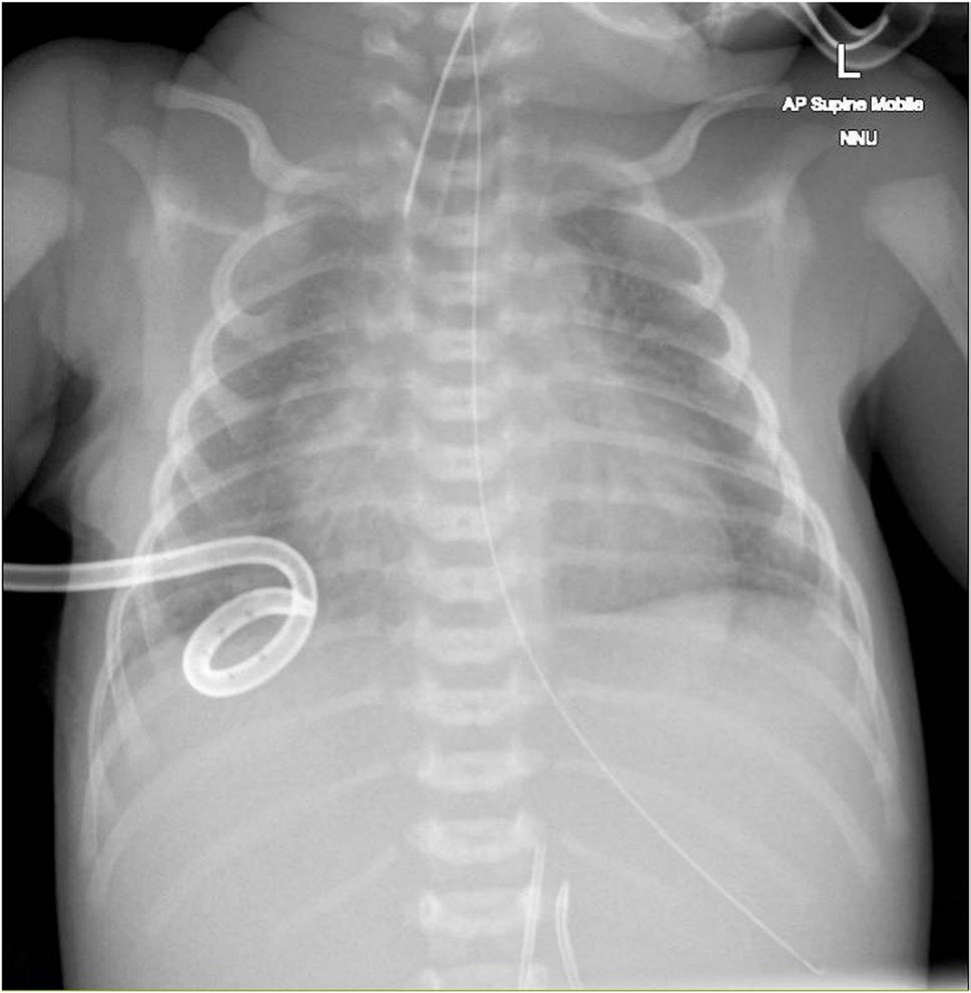
Admission Chest X-ray following chest drain insertion. UVC and UAC were re-inserted due to suboptimal placement

Following this, there was refractory hypotension during the first 24–36h of life, which was managed with dobutamine, noradrenaline, and hydrocortisone. His initial chest X-ray did not reflect appearances of meconium aspiration syndrome (Fig. 1). A bedside echocardiogram on day 1 post-resuscitation revealed poor ventricular contractility, increased right ventricular pressures, and a structurally normal heart. He achieved cardiovascular stability, enabling the weaning of inotropes and nitric oxide before discontinuation on day 3. He was then stable on conventional ventilation with minimal oxygen requirements.

Upon admission, the patient was started on first-line antibiotics (benzylpenicillin and gentamicin), which were later changed to amoxicillin (given the history of meconium-stained liquor in a preterm infant and possible Listeria infection) and gentamicin. His inflammatory markers showed only a modest peak C-reactive Protein (CRP) value of 32.8 mg/L. Aciclovir was added on day three for suspected viral infection due to a significantly elevated alanine aminotransferase (ALT) level (Table 1). He also developed a mild acute kidney injury (Table 1). His blood gas trend demonstrated an initial metabolic acidosis, which was followed by a persistently elevated lactate (> 20mmol/L) on day 3 of life. A baseline metabolic screen was performed, and it was otherwise unremarkable.

**Table 1:** Summary table of patient characteristics

	Case 1	Case 2
Demographics		
Gender	Male	Male
Birthweight (grams)	2407	3400
Gestation (weeks)	35 + 4	37 + 2
Type of delivery	Emergency Caesarean	Elective Caesarean
Day of presentation	1	5
Clinical Features		
Respiratory	**Day 1**: Intubated and ventilated after birth. **Day 1–2**: Right pneumothorax treated with chest drain.	**Day 7**: Intubated and ventilated **Day 11**: Extubated to high-flow nasal cannula oxygen **Day 16**: Self-ventilating in air
Cardiovascular	**Day 1**: Brief cardiac arrest. Adrenaline and dobutamine infusions started. **Day 2**: brief trial of noradrenaline; started on dopamine. **Day 4**: Weaned off inotropic support	**Day 7**: Hypotension – dopamine infusion. **Day 8**: Weaned off inotropic support
Neurological	**Day 1**: normal cranial ultrasound **Day 3**: clinical seizures treated with phenobarbitone **Day 4**: parenchymal haemorrhage on CT	**Day 5**: Lethargy and hypotonia **Day 7**: electrical seizure activity on CFM; treated with phenobarbitone
Investigations		
Lowest haemoglobin (g/L)	105	130
Lowest platelet count (x10^9^/L)	59	10
APTT (seconds)	47.5	77.4
Maximum ALT (U/L)	888	60
Maximum ALP (U/L)	702	308
Maximum CRP (mg/L)	32.8	17.2
Maximum Urea (mmol/L)	18.0	4.1
Maximum Creatinine (µmol/L)	158	43
Enterovirus RNA, blood	Positive (after death; sent on day 3)	Positive (Day 10)
Enterovirus, salivary swab	Positive (Day 4)	Positive (Day 6)
Enterovirus RNA, CSF	Not performed	Positive (Day 10)
Management		
Antimicrobial therapy	Benzylpenicillin (D1) and Gentamicin (D1-D6) Amoxicillin (D1-6) Aciclovir (D3-6)	Benzylpenicillin and Gentamicin (D5) Aciclovir, Cefotaxime, Vancomycin (D6)
Intravenous Immunoglobulin	Day 3	Day 9 and day 11
Blood product transfusions	Packed red cells (Day 1, Day 3) Platelets (Day 3, 4) Cryoprecipitate (Day 3) Fresh frozen plasma (Day 2, 3)	Platelets (Day 6, 11, Day 12) Fresh Frozen Plasma (Day 6)
Final outcome	Death (Day 6)	Discharged home (Day 18)

He was identified as coagulopathic and received treatment with multiple blood products (Table 1). Screening viral salivary swabs tested positive for enterovirus infection. The local tertiary paediatric infectious disease team was consulted and advised that the prevailing diagnosis was disseminated enterovirus sepsis with hepatic failure and disseminated intravascular coagulopathy (DIC). They recommended the administration of IV immunoglobulin, which was subsequently given. Furthermore, the use of pocapavir was advised as a rescue treatment, acknowledging the limited evidence for its benefits in improving the likelihood of survival.

**Fig. 2:**
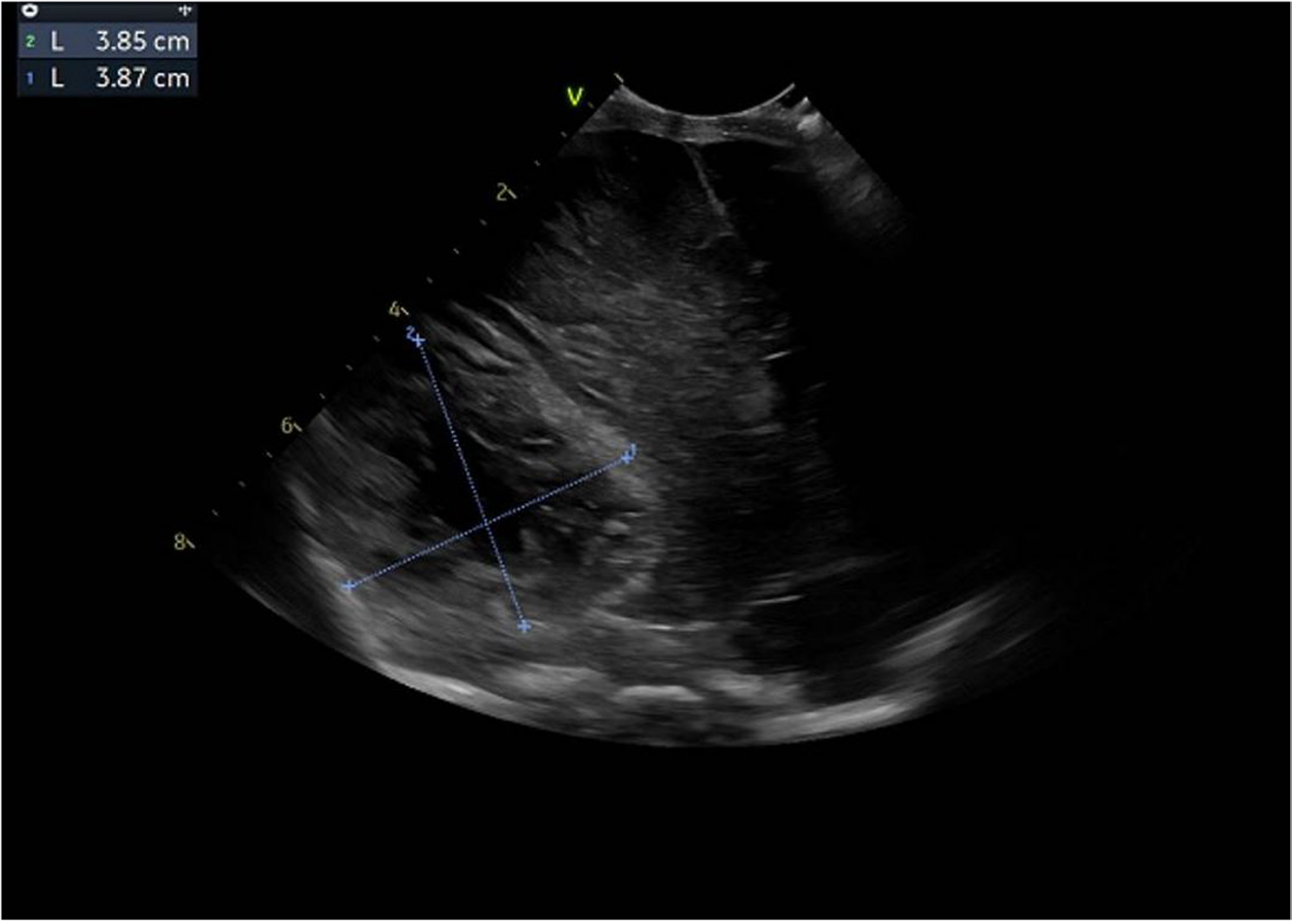
Cranial ultrasound (coronal view) demonstrating suspicious 3.85 × 3.87 cm area indicative of haemorrhage

**Fig. 3:**
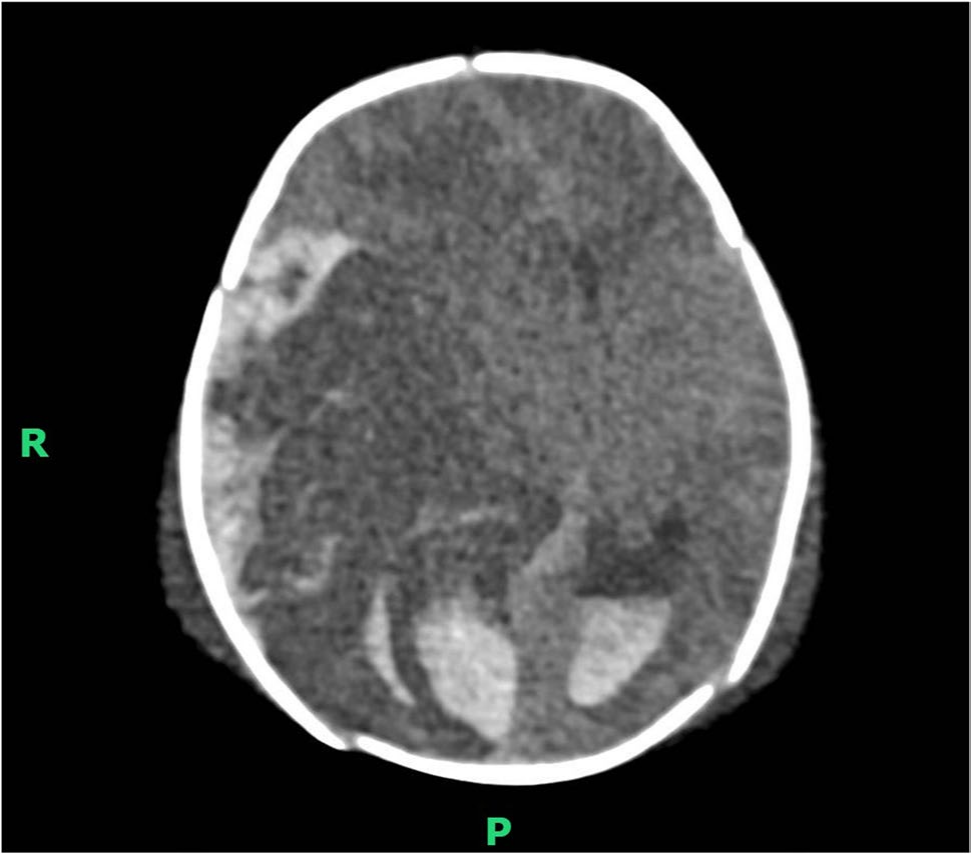
CT Head (axial view) demonstrating extensive haemorrhage and midline shift

He developed clinical seizure activity on day 3 of life. This was corroborated through CFM monitoring, and treatment with phenobarbitone, levetiracetam, and a midazolam infusion was commenced. A cranial ultrasound was suggestive of haemorrhagic infarction with midline shift (Fig. 2), which was confirmed on an urgent CT scan, demonstrating extensive haemorrhage and infarction of both MCA and PCA territories with resulting compression on the midbrain and midline shift (Fig. 3). Additionally, there was parenchymal haemorrhage in the left cerebral hemisphere. The local neurosurgical team concluded that intervention would not be beneficial and suggested palliative care, anticipating a poor prognosis. Pocapavir was not administered due to the expected withdrawal of care. After considering the patient’s poor likelihood of survival and high risk of significant neurodisability, care was subsequently redirected following discussions with his family. Sadly, he passed away on day six. The serum PCR for enterovirus returned a positive result after his death, confirming severe neonatal enterovirus infection.

### Case B

**Fig. 4:**
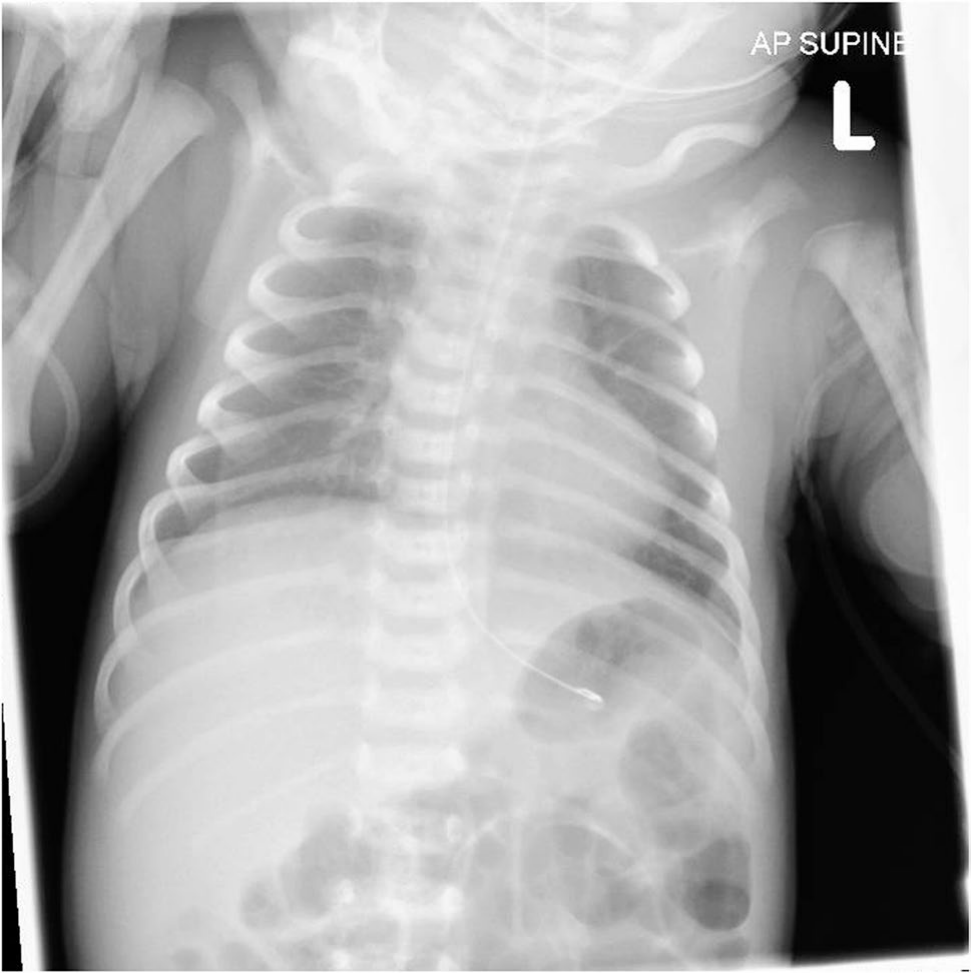
Admission Chest X-ray

A male infant weighing 2400 g was born at 37 + 2 weeks via elective Caesarean section in a local district general hospital. He cried immediately after birth, with Apgar scores of 9 at 1 min, 10 at 5 min, and 10 at 10 min. There were no complications during the pregnancy, although his mother had experienced mild rhinorrhoea and coryza at the time of birth. Both mother and infant were discharged home on day 3 of life. On day 5, his mother was re-admitted with a post-operative wound infection when the infant was incidentally found to be intermittently grunting. On further examination, the infant was lethargic and mildly hypotonic with a low-grade fever. After a brief episode of desaturation, he was admitted to the neonatal unit and commenced on empirical first-line antibiotics (Table 1). Initial blood tests and chest X-ray (Fig. 4) were unremarkable, showing platelets of 165 × 10^9^/L and a CRP of 5 mg/L, while renal and liver function tests remained within normal ranges.

Twelve hours after admission, on day 6, repeat blood tests revealed an evolving profound thrombocytopenia and coagulopathy (Table 1) alongside a rising CRP (17 mg/L), necessitating multiple blood product transfusions (Table 1). Antibiotic therapy was escalated to second-line treatment (cefotaxime, vancomycin) and aciclovir was added due to abnormal liver function, with suspicions of viral infection. A TORCH screen was negative. Feeds were withheld, and he was kept nil by mouth.

On day 7, he was intubated following multiple episodes of apnoea with an increased oxygen requirement. Cerebral Function Monitoring (CFM) demonstrated seizure-like activity that responded to 20 mg/kg of phenobarbitone. The infant was transferred to a tertiary neonatal intensive care unit, where peripheral dobutamine was started for 24 h to treat transient hypotension. An initial neurological assessment at the tertiary centre revealed hypertonia and a poor suck reflex. Serum troponin (963ng/L) and ferritin levels (2,468 µg/L) were elevated, raising suspicion of macrophage activation syndrome. An echocardiogram was normal, and cranial ultrasounds were normal throughout. CFM monitoring was continued for 72 h with no further episodes of seizures.

A viral respiratory swab collected on day six subsequently tested positive for Enterovirus, which was confirmed by a positive serum PCR sample. Enterovirus was also detected in cerebrospinal fluid following a lumbar puncture on day 10 of life. After discussions with the infectious diseases team, the infant received intravenous immunoglobulin on days 9 and 11 of life. Pocapavir was discussed but not administered as the baby had shown clinical improvement by this time.

Enterovirus was never confirmed in maternal samples, while all other microbiology and metabolic investigations were negative. Ventilation was uncomplicated and the infant was successfully extubated on day 11 of life. Four additional platelet transfusions were administered to achieve stabilisation. Enteral feeds were introduced via nasogastric tube and breastfeeding was tolerated. The baby was discharged home on day 18 of life. Follow-up at four months did not reveal any neurological or developmental concerns or thrombocytopenia. An EEG at two months of age demonstrated no further epileptiform activity.

## Discussion

In the United Kingdom, the incidence of neonatal enteroviral infections has been estimated as 0.79 per 1,000 live births [3]. Several serotypes are associated with neonatal illness, including echovirus (EV) 11 and 25, as well as coxsackievirus (CV) B1-4 [4]. There can be seasonal patterns of incidence, with one recently documented outbreak in the United Kingdom between 2022 and 2023 involving 30 neonatal cases, of which 16 required intensive care [5]. These infections can present with non-specific symptoms and may be recognised late, with estimated detection rates of enterovirus among children presenting with sepsis-like illnesses ranging from 24% to 37% [6, 7].

Several potential routes of infection have been proposed, supported by evidence of vertical transmission through the occurrence of early-onset infections [8] and the isolation of enterovirus from amniotic fluid [9]. This is often associated with a period of acute illness approximately one week prior to birth [10]. Nosocomial transmission has also been well established, with reports of outbreaks in neonatal units [8, 11]. Early infection has been linked to abnormalities on magnetic resonance imaging (MRI) of the brain [12].

Clinical manifestations of neonatal enteroviral infections are classically non-specific, including symptoms such as temperature instability, rash, poor feeding, lethargy and jaundice [1]. The most recognised severe complication is myocarditis, associated with CVB1-5 infections, which often presents with arrhythmias, circulatory failure or poor perfusion [1]. Up to 66% of survivors may develop serious chronic sequelae such as heart failure, aneurysm formation and mitral regurgitation [13]. Additionally, fulminant hepatitis and coagulopathy can also occur, often in association with the CVB and EV 11 serotypes [14]. In these cases, thrombocytopenia, prolonged clotting times, elevated transaminase values and hyperbilirubinaemia can be observed, with a risk of haemorrhagic complications, resulting in an estimated case fatality rate of 31% [15]. Lastly, meningoencephalitis can also occur, presenting with lethargy, seizures, hemiparesis, altered consciousness or bulging fontanelles [1]. It is the most common cause of viral meningitis in young children, with an incidence twice that of bacterial meningitis [3], though it is somewhat rarer in neonates [1].

Suggested investigations for suspected neonatal enterovirus include cerebrospinal fluid (CSF), stool, blood, and respiratory samples for PCR analysis [16]. Combined PCR analysis of both CSF and serum has been observed to be more sensitive than either method alone [17]. Cerebrospinal fluid pleocytosis may be observed in children with enteroviral infections [7].

In both cases of disseminated neonatal enteroviral infections, there was a cluster of symptoms with significant confounding factors creating diagnostic uncertainty. Both patients initially presented with respiratory distress, which rapidly progressed to multiorgan failure and DIC. To the best of our knowledge, PPHN and neonatal stroke have not previously been described in cases of neonatal enterovirus. Neonatal stroke, meanwhile, is most likely related to severe coagulopathy.

The two cases also demonstrate the rapid potential for deterioration in enteroviral sepsis. Despite being identified following an incidental finding of respiratory distress on the postnatal ward, within 48h in Case B, the baby was ventilated with progressive coagulopathy and electrical seizure activity. There was also a history of brief maternal illness, which may suggest peripartum enteroviral infection. Both patients required multiple blood products to ensure stabilisation.

The outcomes in both cases differed due to the severity and extent of neurological involvement. In Case A, the cardiorespiratory condition improved but the development of a neonatal stroke was ultimately fatal. In Case B, despite the occurrence of meningitis, there were no lasting neurological complications, resulting in a complete recovery.

Treatment of severe cases is primarily supportive. The main treatment of choice is intravenous immunoglobulin (IVIG), which has been linked to favourable outcomes in cases of severe enteroviral infections, albeit within small study populations. One study of 16 neonates reported significantly higher serum neutralising titres following IVIG administration [18], while another study of 67 enteroviral hepatitis cases observed that early IVIG administration was associated with a favourable prognosis [19]. This is particularly important, as non-specific symptoms may be erroneously attributed to bacterial infections due to diagnostic bias, and obtaining intravenous immunoglobulin promptly may be challenging.

There are no licensed antiviral medications for use in neonatal enteroviral infections. Plecoranil, a capsid inhibitor, was investigated in a randomised controlled trial of 61 neonates, which observed shorter times to culture and PCR negativity, although these results were not statistically significant [20]. Plecoranil has not received FDA approval due to concerns related to CYP3A enzyme activity. Pocapavir, which was considered in this case, is another capsid inhibitor which yields some promise with case reports describing favourable outcomes following treatment in severe enteroviral sepsis, including reductions in enterovirus titres post- treatment [21], improvements in cardiac function following myocarditis [22], improved coagulopathy and resolved multi-organ dysfunction [23, 24]. Nevertheless, this has not been proven in larger clinical trials and is limited to experimental or compassionate settings.

Extra-corporeal membrane support (ECMO) can prevent further deterioration and cardiac arrest in neonates affected with severe myocarditis, as highlighted in a retrospective case series of 7 infants testing positive for enterovirus within a paediatric intensive care unit [25]. Another review of 24 ECMO patients with neonatal enterovirus infection identified multiorgan dysfunction and renal failure as poor prognostic indicators [26]. In our case, escalation to ECMO was considered from a respiratory perspective, but was not required as stabilisation was achieved following treatment for a pneumothorax.

Lastly, follow-up data is limited, with outcomes ranging from no overt sensorineural deficits [3] to significantly lower Bayley III cognitive scores, albeit within normal ranges [12]. Pathological findings have also been identified on neuroimaging of neonates with enteroviral infections, including dural contrast enhancement, diffuse white matter abnormalities, periventricular bleeds and cytotoxic oedema [12].

These cases highlight the key diagnostic challenges in identifying neonatal enteroviral infections and emphasise the importance of maintaining a broad differential diagnosis, including viral infections. While coagulopathy and meningitis are well-known complications, severe respiratory compromise and haemorrhagic strokes have rarely been described in the literature. Early recognition of enterovirus can help to manage expectations and facilitate the administration of IVIG, which may confer a survival benefit. In conclusion, these cases demonstrate that marked hypoxia, coagulopathy and hepatic or metabolic dysfunction inconsistent with the clinical picture should raise suspicion of viral infections, whilst haemorrhagic stroke and PPHN should be considered as separate manifestations of neonatal enterovirus.

## Data Availability

Not applicable – no datasets were generated. All relevant clinical data pertaining to the case series are included in this published article.

## Abbreviations

PPHN: Persistent pulmonary hypertension of the newborn
CRP: C-reactive protein
CFM: Cerebral function monitoring
ALT: Alanine aminotransferase
ALP: Alkaline phosphatase
FFP: Fresh frozen plasma
PTT: Partial thromboplastin time
APTT: Activated partial thromboplastin time
PCR: Polymerase chain reaction
IV: Intravenous
CT: Computed tomography
RNA: Ribonucleic acid
MCA: Middle cerebral artery
PCA: Posterior cerebral artery
TORCH: Toxoplasma, other, rubella, cytomegalovirus, herpes simplex virus
DIC: Disseminated intravascular coagulopathy
UAC: Umbilical arterial catheter
UVC: Umbilical venous catheter
